# Childhood Adversity Impairs Theory of Mind Abilities in Adult Patients With Major Depressive Disorder

**DOI:** 10.3389/fpsyt.2019.00867

**Published:** 2019-12-17

**Authors:** Maria Simon, Nándor Németh, Mónika Gálber, Elza Lakner, Eszter Csernela, Tamás Tényi, Boldizsár Czéh

**Affiliations:** ^1^Neurobiology of Stress Research Group, János Szentágothai Research Center, University of Pécs, Pécs, Hungary; ^2^Department of Psychiatry and Psychotherapy, Clinical Center, Medical School, University of Pécs, Pécs, Hungary; ^3^Department of Laboratory Medicine, Clinical Center, Medical School, University of Pécs, Pécs, Hungary

**Keywords:** adverse childhood experiences, depression, childhood trauma questionnaire, early-life stress, mental state decoding, reading the mind in the eyes test, theory of mind

## Abstract

**Background:** Patients with major depressive disorder (MDD) have various theory of mind (ToM) impairments which often predict a poor outcome. However, findings on ToM deficits in MDD are inconsistent and suggest the role of moderating factors. Child abuse and neglect are strong predictors of adult MDD and are often associated with a poorer clinical course trajectory.

**Objective:** Because early-life adversities result in various forms of ToM deficits in clinical and nonclinical samples, our aim was to investigate if they are significant confounding factors of ToM impairments in MDD.

**Methods:** We investigated 60 mildly or moderately depressed, nonpsychotic adult patients with MDD during an acute episode, and 32 matched healthy controls. The mental state decoding subdomain of ToM was examined with the Reading the Mind in the Eyes Test (RMET). Childhood adversities were assessed with the childhood trauma questionnaire (CTQ) and the early trauma inventory.

**Results:** There was no difference between the control and MDD groups in RMET performance. However, when we divided the MDD group into two subgroups, one (N = 30) with high and the other (N = 30) with low levels of childhood adversities, a significant difference emerged between the controls and the highly maltreated MDD subgroup in RMET performance. A series of 3 (group) × 3 (valence) mixed-model analyses of covariance (ANCOVAs) revealed that childhood emotional and physical neglect had a significant negative impact on the response accuracy in RMET in general, whereas emotional abuse specifically interfered with the accuracy in the positive and negative valences if it co-occurred with early-life neglect. To test the dose-response relationship between the number of childhood adversities and RMET capacities, we subjected RMET data of the MDD group to multiple hierarchical regressions: the number of childhood adversities was a significant predictor of RMET total scores and RMET scores in the negative valence after controlling for age, sex, years of education, and the severity of current depression.

**Conclusion:** Childhood adversities impair ToM capacities in MDD. Exposure to early-life emotional abuse and neglect have a negative impact on the performance in the emotional valences of RMET. Multiple early-life adversities have a dose-dependent association with mental state decoding deficits.

## Introduction

Patients with major depressive disorder (MDD) have various impairments in social functioning, e.g., withdrawal from social interactions, impaired social competence, negative interpersonal experiences, as well as less enjoyment in social relations (1). Depressed patients can feel self-absorbed, detached from their environment, as well as not knowing how to approach others (2). Typically, the low level of social functioning in patients with depression can be ascribed to social cognitive deficits. Theory of mind (ToM) is one of the essential components of social cognition: the ability to infer the mental states of others by representing their beliefs, intentions, desires, and fears. Hence, ToM is the capacity to attribute mental states (i.e., beliefs, desires) to self and other people, and to understand and predict their behaviors, intentions, and wishes (3). Although the findings are inconsistent, numerous studies detected that patients with MDD have ToM impairments. A recent meta-analysis aggregating 18 clinical studies demonstrated that patients with MDD significantly underperformed healthy controls in different types of ToM tasks (4).

ToM is a multidimensional construct involving several dimensions. Sabbagh (5) identified two components of ToM: (1) the socioperceptual component or mental state decoding: the ability to detect and discriminate cues in the immediate social environment, i.e., the ability to *decode* others’ mental states; and (2) the sociocognitive component or mental state reasoning: the ability to infer about social cues, i.e., the ability to *reason* about the mental states of others. Hence, mental state decoding is the initial step of ToM that requires various socioperceptual skills, i.e., adequately identifying and differentiating emotional facial expressions, whereas mental state reasoning abilities encompass sociocognitive capacities, i.e., recognizing conversational failures, as well as interpreting others’ emotional states or thoughts.

The Reading the Mind in the Eyes Test (RMET, 6) is a widely accepted ToM test to measure mental state decoding (i.e., the socioperceptual component of ToM). In RMET, participants view a series of black-and-white photographs of the eye region of actors’ faces, and they are instructed to judge which of the four adjectives presented simultaneously with the eyes best describe the emotional state of the person in the picture. RMET presents subtle emotional information that embraces a relatively wide range of mental states beyond the basic emotions. Requiring no inferences about cognitive and affective mental contents, as well as no contextual processing, RMET can be regarded as an appropriate task to measure the initial, decoding (or discriminating) ToM processes, predominantly the decoding of subtle facial affective cues. On the basis of the emotional content of the target adjective, RMET photographs have been categorized into three valence groups: neutral (e.g., reflective, thoughtful), negative (e.g., panicked, jealous, hateful), as well as positive (e.g., friendly, playful) (7).

So far, several studies have examined the accuracy of mental state decoding in RMET in clinical and population samples with depressive symptoms as well as in individuals with increased vulnerability to depression (7–13). However, the findings were often controversial, which implies the role of moderating factors such as symptom severity, clinical phase, and feature (including psychotic symptoms, chronicity, as well as acute vs remitted depression). A recent meta-analysis involving seven studies on mental state decoding reported that patients with MDD performed significantly less accurate in RMET (14). Because of the lack of data on other clinical variables and biographic factors, this meta-analysis considered only the effect of psychiatric comorbidities. Nevertheless, the role of early adverse experiences or traumatic childhood events in ToM deficits of depressed patients remained understudied.

However, the negative impact of childhood maltreatment on ToM capacities has been detected in various clinical and nonclinical community samples, e.g., in maltreated youth (15, 16), in a large internet-based, population sample (17), in patients with borderline personality disorder (18, 19) as well as in female patients with post-traumatic stress disorder (PTSD) (20). Moreover, adverse childhood experiences (e.g., physical, emotional, and sexual abuse, neglect, parental loss, and poverty) have long been known to be strong predictors of adult MDD (e.g., 21–23), and early-life adversities were associated with a more severe and persistent course of MDD (24–26). On the other hand, a growing number of data suggests that ToM abnormalities predict relapses and a worse outcome in MDD due to the difficulties in social adjustment (27,). Yet, hardly any studies examined the effect of traumatic life events experienced during childhood on mental state decoding abilities in patients with MDD, so far. Merely, a very recent study reported that childhood emotional abuse was associated with poorer response accuracy in RMET in adult patients with MDD, while physical abuse negatively influenced the mental state decoding accuracy of healthy, never-depressed controls (29). In addition, neglect was associated with poorer RMET accuracy across both groups. Although this groundbreaking study focused on the effect of the different forms of childhood adversities, it did not consider the general effect of early-life stress and the effect of multiple adversities on RMET accuracy.

On the basis of the literature, we designed a study in which the mental state decoding component of ToM was assessed in a relatively homogeneous group of MDD patients. We examined the mental state decoding (RMET) capacities in patients with acute, nonpsychotic MDD and in healthy, never-depressed, population controls. We assessed the severity of childhood adversities and then examined their impact on the RMET performance of depressed patients. To characterize the effect of childhood adversities further, we also evaluated the effect of physical, emotional, sexual abuse, and neglect on the response accuracy across the various RMET valences. We hypothesized that patients with depression will have poorer RMET accuracy than healthy controls, and that adverse childhood experiences will have a worsening effect on these deficits.

## Methods

### Participants

Patients with MDD (N = 60) were recruited from the affective disorder unit of the Department of Psychiatry and Psychotherapy, University of Pécs. All patients fulfilled the DSM-5 diagnostic criteria of MDD (30). Inclusion criteria of the MDD group included: (1) age 18–55 years; (2) a diagnosis of MDD in a current major depressive episode (≥8 points on the Hamilton Depression Rating Scale (HAM-D), 21-item version (31). Because the clinical sample was recruited from the acute setting, the relatively long screening and testing procedure did not make it possible to investigate severely depressed participants. Exclusion criteria of the patient group were: current substance abuse or dependence (if the patient met diagnostic criteria he or she had to be abstinent for at least 2 years); current and lifetime psychotic symptoms, bipolar disorder, organic psychiatric disorders. Patients with any history of severe internal medical or neurological disorders, in addition, those with a history of head injury and with severe hearing or visual impairment; and an Intelligence Quotient (IQ) < 85 were also excluded. Although MDD is a common comorbid disorder in borderline personality disorder, considering the controversial data on RMET performances in patients with borderline personality disorder (meta-analyzed by 14,), MDD patients with comorbid borderline personality disorder were also excluded from this study.

The mean age of disease onset was 24.81 (±8.83) years. The mean duration of illness was 9.13 (±7.53) years with a range of 0.4–26 years. Fifty-seven (95%) patients with MDD received antidepressant medication (SSRI: 29; SNRI: 5; NaSSA: 12; bupropion: 2, agomelatine: 5, trazodone: 2; in combination with mood stabilizer: 3; in combination with low-dose atypical antipsychotic: 3).

The healthy control group (HC, N = 32) was matched in age, sex, and level of education. HCs were screened by a qualified psychiatrist to ascertain the absence of lifetime or family history of mental disorders; in addition, SCL-90 (33) was applied to rule out relevant subthreshold psychiatric symptoms in the potentially healthy individuals. Exclusion criteria for controls included a history of substance abuse in the past 24 months, a history of neurological disorders, a history of head injury with loss of consciousness for more than 30 min, an IQ < 85, and any learning difficulties. None of the healthy individuals took psychotropic medication. Four participants of the HC reached scores in the childhood trauma questionnaire (CTQ) in the moderate range and consequently, they were excluded from the study.

The local Research Ethics Committee of the University of Pécs approved the study design and protocol (Ethical Approval Nr.: 2015/5626) and all participants provided written informed consent.

### Clinical Assessments

1. Diagnostic assessment: the current major depressive episode was assessed by a trained psychiatrist using the Structured Clinical Interview for DSM-5 disorders (SCID-5-CV and SCID-5-PD; 34, 35). Patients with MDD had nonexcluded comorbidities as follows: anxiety disorders (panic N = 7; social phobia N = 5; GAD N = 3; specific phobias N = 2), PTSD (N = 1), current OCD (N = 1), lifetime OCD (N = 2), lifetime alcohol use disorder (N = 3), lifetime sedatives, hypnotics, and anxiolytics use disorder (N = 4); cluster C personality disorders (dependent N = 4, avoidant N = 2).
2. Depression severity was evaluated using a multimethod approach. A trained clinician completed the 21-item Hamilton Rating Scale for Depression (HAM-D). The Beck Depression Inventory (BDI; 36) was applied as a self-assessment inventory.
3. Clinical data in patients with MDD (i.e., age at onset, length of illness, number of episodes, medications) were collected by clinical interviews, as well as by reviewing the affective disorder unit’s charts and in- or outpatients’ files.

### Intelligence Quotient (IQ)

Full-scale IQ was measured using the Hungarian version of the Wechsler Adult Intelligence Scale (37, 38). Total IQ, performance intelligence quotient (PQ), and verbal intelligence quotient (VQ) values were calculated.

### Assessment of Childhood Maltreatment

1. A senior psychiatrist (MS) conducted a structured interview with a focus on childhood adversities with all participants. Participants’ answers during the interview were recorded on a preprinted interview sheet.
2. Childhood maltreatment was surveyed with the 28-item retrospective self-report questionnaire: the CTQ-short form (39) that assesses the severity of five types of maltreatment before the age of 18 years: physical abuse (PA), emotional abuse (EA), physical neglect (PN), emotional neglect (EN), and sexual abuse (SA). Each subscale consists of five items, all of them are evaluated on 5-point Likert scales. In our department, the internal consistency was excellent for the subscales: EA = 0.93, EN = 0.94, SA = 0.97, PA = 0.93, and good for the subscale PN = 0.77. During the data analysis, CTQ raw scores were recoded into a two-level, binary variable by cut-off values on each subscale. Cut-off values were defined on the basis of a large normative sample consisting of 330 participants (university students and community sample).
3. Traumatic childhood experiences were also quantified with the self-report form of the Early Trauma Inventory—Self-Report (ETI) questionnaire (40). This 27-item questionnaire has 11 items for general traumatic experience, 5 PA items, 5 EA items, and 6 SA items that may have occurred before the age of 18 years. Each item can be answered with “yes” (scored as 1) or with “no” (coded as 0). For general traumas, factor analysis found three factors from which scores of the “dysfunctional family events” subscale (including witnessing family violence, separation of the parents, alcoholic parents) were entered into the further analyses.
4. Finally, interview results were compared with scores on trauma scales by a psychologist blinded to the patient. Discrepancies were discussed with the participants. In the case of unresolvable discrepancies, the participant was excluded from further analyses (N = 3).

On the basis of the measures listed above, the following variables were derived for further analysis:

1. Examining the impact of the prevalence of any trauma: when CTQ scores in any trauma dimension were at least in the moderate range, then, exposure to high childhood adversities (ACE) was assumed. Thus, patients were assigned to the *high-ACE MDD* subgroup if they had at least one type of moderate to severe child abuse and patients who did not experience any moderate to severe child abuse formed the *low-ACE MDD* subgroup.
2. Examining the impact of the specific traumas: All specific trauma measures were derived as binary measures from the scores of specific subscales (EN, EA, PN, PA, SA) of CTQ-SF. It was one if the scores were at least in the moderate range. Hence, patients with MDD were divided into *high-* and *low-EN, EA, PN, PA, and SA subgroups*.
3. Examining the impact of cumulative trauma: The variable “*number of traumas*” was calculated as a sum of the binary measures of specific types of adversities in CTQ and the “dysfunctional family events” score of the ETI. (Dysfunctional family score was one if the participant answered all of the three items with “yes.”)

### Assessment of ToM

Mental state decoding capacities were assessed using the revised version of the RMET (6). In RMET, a series of black-and-white photos presenting only the eye region is shown, and participants are instructed to pick one from four words presented simultaneously with the eyes to describe best the emotional state of the person in the photo. RMET consists of 36 trials and has been proven to be a valid measure of social sensitivity or mindreading (= mental state decoding). It shows a good test–retest reliability with no ceiling effect. Response accuracy and response time (in milliseconds) were digitally recorded. Based on Harkness et al. (7), items of the RMET were classified according to the emotional valences as positive (e.g., “friendly,” N = 8), negative (e.g., “despondent,” N = 12), or neutral (e.g., “pensive,” N = 16).

### Data Collection Procedures

During the first session, participants underwent diagnostic assessment and demographic interviews, then traumatic childhood experiences were assessed. In the second session (within 2 days), participants completed the RMET.

### Statistical Analysis

First, initial exploratory *t* tests, chi-square tests, as well as bivariate correlations were conducted to explore associations between demographic and clinical variables, as well as ToM performances and the severity of childhood trauma. Subsequently, one-way ANCOVAs were performed to test between-subject differences in RMET scores with age, sex, and years of education as covariates.

Then, a series of 3 (group) × 3 (valence) mixed-model ANCOVAs with age, sex, and years of education as covariates was performed. To examine the effect of specific childhood adversities on the RMET accuracy, MDD patients were grouped on the basis of the specific subscales of the CTQ. First, RMET data of HC, as well as low- and high-ACE MDD groups were compared with an ANCOVA with age, sex, and years of education as covariates. Then, RMET data of healthy subjects and those of MDD patients with high- and low-EN, EA, PN, PA, as well as SA were entered into 3 (group) × 3 (valence) mixed-model multivariate ANCOVAs with age, sex, and years of education as covariates. Because of the overlap between the prevalence of some types of traumas, further multivariate ANCOVAs with controlling for other traumas were also performed.

To test the dose-response relationship between the cumulative effect of childhood adversities and the RMET performance across the various valences within the MDD group, the variable “number of traumas” was calculated. RMET total accuracy, as well as accuracies in the various valences, were entered as outcome variables in hierarchical multiple regression models. Predictor variables were entered in three steps: first, the predictive effect of the demographic variables (age, sex, and years of education) was tested, then, the severity of depression (i.e., BDI score) was added to the model, last the “number of traumas” was entered. Multicollinearity was tested with variance inflating factor (VIF), effect sizes were measured with η^2^. The level of significance was set at *p* = 0.05.

## Results

### Description of the Sample

Demographic and clinical variables, as well as IQ scores, are shown in **Tables 1** and **2**. Initial exploratory analyses (chi-square and *t* tests, as well as bivariate correlations, a series of ANOVAs) were conducted to find covariates for multivariate models (see the **Supplementary Material**).

**Table 1:** Demographic and IQ data of the sample: there were no significant between-group differences.

	HC N = 32	MDD N = 60	2-group comparison statistics	low-ACE MDD N = 30	high-ACE MDD N = 30	3-group comparison statistics
Age (SD)	32.97 (7.75)	32.91 (8.39)	*t*_(90)_ = 0.3	33.15 (7.56)	32.7 (9.07)	*F*_(2,89)_ = 0.02
Females N (%)	20 (66%)	42 (75%)	?^2^_(1)_ = 0.67	19 (73%)	23(%)	?^2^_(2)_ = 0.76
Years of education (SD)	13.85 (2.15)	13.48 (2.49)	*t*_(90)_ = 0.62	14 (2.24)	13.10 (2.71)	*F*_(2,89)_ = 1.28
Tertiary education N (%)	14 (46.7%)	24 (42.85%)	?^2^_(1)_ = 0.12	13 (50%)	11 (36.7%)	?^2^_(2)_ = 1.12
IQ	111.6 (6.2)	110.4 (4.96)	*t*_(90)_ = 1.31	110.9 (4.79)	109.9 (5.14)	*F*_(2,89)_ = 1.45
VQ	110.5 (8.32)	111.7 (3.82)	*t*_(90)_ = 0.71	112.7 (5.08)	110.8 (7.31)	*F*_(2,89)_ = 2.68
PQ	112.7 (8.52)	109.6 (6.58)	*t*_(90)_ = 1.86	109.8 (7.39)	(109.4 (5.91)	*F*_(2,89)_ = 1.42

HC, healthy control group; MDD, Major depressive disorder group; ACE, adverse childhood experiences; high-ACE MDD, a subgroup of MDD patients with at least one type of any abuse or neglect in the moderate or severe range; low-ACE MDD, a subgroup of MDD patients with no abuse or neglect in the moderate or severe range. VQ, verbal intelligence quotient, PQ, performance intelligent quotient.

**Table 2:** Clinical variables of the sample.

	HC N = 32	MDD N = 60	2-group comparison statistics	low-ACE MDD N = 30	high-ACE MDD N = 30	3-group comparison statistics
HAM-D (SD)	2.39 (1.54)	16.63 (2.71)***	*t*_(90)_ = 27.99	15.88 (2.2)	17.27 (2.97)	K-W stat = 2.81
BDI (SD)	4.43 (2.75)	23.02 (4.46)***	*t*_(90)_ = 20.8	22.23 (3.26)	24.7 (5.7)^+^	*F*_(2,89)_ = 219.7*** *post hoc*: HC< lowACE MDD < highACE MDD
BAI (SD)	6.13 (5.87)	20.05 (8.27)***	*t*_(90)_ = 8.47	19 (8.75-24)^a^	23.5 (16.75-27.25)^a++^	K-W stat = 47.41*** *post hoc*: HC < low-ACE MDD < high-ACE MDD
Age at onset (SD)	–	25 (17-39)	–	27 (19.13-32.25)	19 (16-19.13)	*U* = 322
Length of illness (years)^a^	–	8 (5.25-12)	–	8.5 (6-12)	8.0 (4.5-10)	*U* = 331
Recurrent depression N (%)	–	33 (55%)	–	12 (46.15%)	23 (76.6%)	?^2^_(2)_ = 6.94
Number of episodes	–	2 (2)^a^	–	1 (1-3)^a^	2 (1-5)^a++^	*U* = 255
AD medication Yes (%)	–	58 (96.7%)	–	28	29	?^2^_(2)_ = 0.318

^a^ medians (interquartile intervals) are presented; ***p < 0.001 compared to the HC; + p < 0.05 compared to the low-ACE MDD group; ++ p < 0.01 compared to the low-ACE MDD group.

HC, healthy control group; MDD, Major depressive disorder group; ACE, adverse childhood experiences; high-ACE MDD, MDD patients with at least one type of abuse or neglect in the moderate or severe range; low-ACE MDD, MDD patients with no abuse or neglect in the moderate or severe range. K-W stat, Kruskal-Wallis statistic; U, Mann-Whitney U.

### Child Abuse and Trauma Questionnaire Scores

Twenty-five MDD patients reported multiple adversities (41.67%), seven patients reported 2 (11.67%), eight 3 (13.33%), seven 4 (11.67%), and three 5 (5%) at least a moderate degree of childhood adversity. **Table 3** gives an overview of the frequency of the various forms of childhood adversities. A substantial co-occurrence of the specific types of childhood adversities was detected. Emotional abuse was strongly correlated with physical traumas and emotional neglect. The most common form of early-life adversity was emotional neglect followed by emotional abuse (95% of the emotionally neglected patients were also emotionally abused). All sexually abused patients were emotionally neglected, and 91.7% of them were at least moderately emotionally abused. 86% of the physically neglected MDD patients were emotionally neglected, and 73.3% of them emotionally abused. Physical abuse was relatively rare, but emotional neglect and emotional abuse nearly always accompanied it (in 91% and 83% of the cases, respectively). Spearman’s correlation analyses revealed that RMET total scores significantly correlated with CTQ total scores (rho = –0.232), CTQ PA (rho = –0.220), EA (rho = –0.234) EN (rho = –0.214), as well as RMET negative scores were significantly correlated with CTQ PA scores (rho = –0.269).

**Table 3:** RMET data: comparison of healthy controls with patients with MDD.

	HC N = 32	MDD N = 60	ANOVA	ANCOVA^a^
RMET total score	74.13 (8.51)	70.46 (9.8)	*F*(_1,91_) = 3.18 *ns*	*F*(_1,88_) = 3.29 *ns*
RMET neutral valence	77.73 (12.49)	72.92 (11.83)	*F*(_1,91_) = 3.33 *ns*	*F*(_1,88_) = 3.03 *ns*
RMET positive valence	81.25 (16.50)	78.54 (16.61)	*F*(_1,91_) = 0.56 *ns*	*F*(_1,88_) = 1.00 *ns*
RMET negative valence	64.84 (10.94)	61.81 (14.82)	*F*(_1,91_) = 1.04 *ns*	*F*(_1,88_) = 0.92 *ns*

^a^Analysis of covariance with age, sex, and years of education as covariates. ns, not significant. Data are presented as mean (standard error). All RMET data are expressed as percent of the accurate answers.

HC, healthy control group; MDD, Major Depressive Disorder group; RMET, Reading the Mind in the Eyes Test.

### Mental State Decoding Accuracy: RMET Performances

#### RMET Accuracy in HC Versus Overall MDD Group

There were only trend level differences between the HC and MDD groups in the overall RMET accuracy (RMET total) scores (*p* = 0.077, η^2^ = 0.034) and in RMET scores in neutral valences (*p* = 0.071, η^2^ = 0.036). This between-group difference did not substantially change when controlling for age, sex, and years of education in the ANCOVA with overall RMET scores (*p* = 0.073, η^2^ = 0.036) and with RMET scores in neutral valences (*p* = 0.085; η^2^ = 0.034). (**Supplementary Material** presents all statistics of between-group comparisons of the RMET data.)

#### The Effect of Childhood Adversities on ToM Performance in MDD

**Figure 1:**
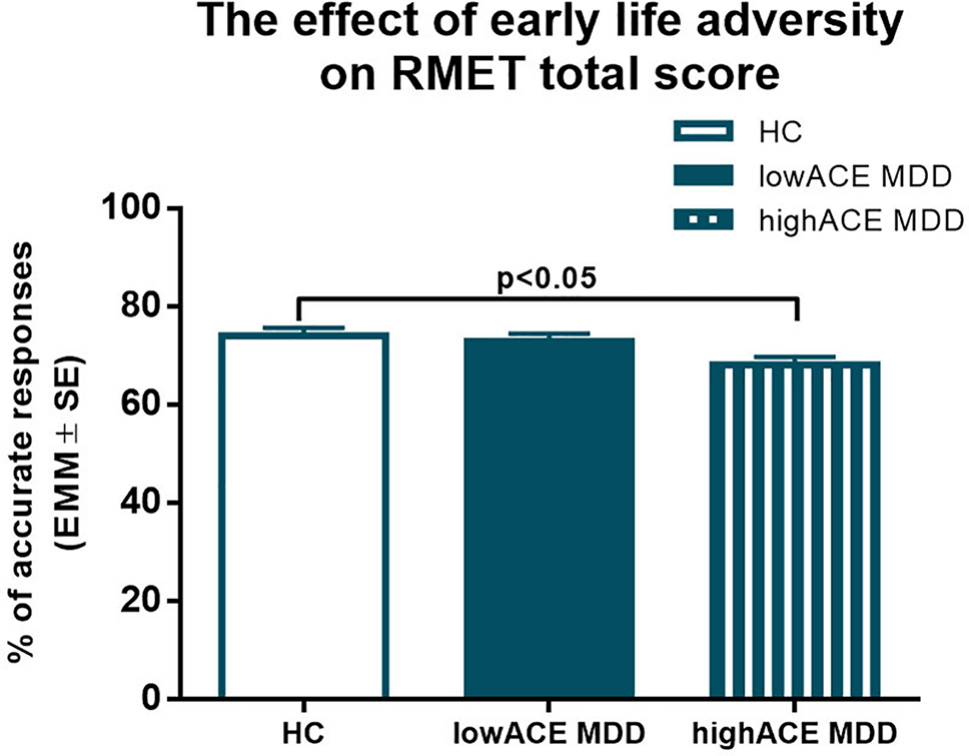
Results of one-way ANCOVA with age, sex, and years of education as covariates. Estimated marginal means (EMM) are presented. Post hoc Bonferroni correction revealed that MDD patients with at least one type of moderate or severe childhood adversity performed significantly worse in RMET compared to healthy controls. ACE: Adverse Childhood Experience, HC: healthy control group, LowACE MDD: a subgroup of MDD patients without any moderate or severe childhood abuse or neglect, HighACE MDD: a subgroup of MDD patients with at least one type of moderate or severe childhood abuse or neglect.

One-way ANCOVA was conducted to determine a statistically significant difference between HC, low-ACE, and high-ACE MDD groups on RMET total scores controlling for age, sex, and years of education. There was a significant effect of group on RMET performance [*F*_(2,86)_ = 3.87, *p* = 0.025, η^2^ = 0.083]. Post hoc Bonferroni correction indicated that the RMET total score of the high-ACE MDD group was significantly lower than that of the HC (**Figure 1**).

##### The Effect of Specific Types of Adversities on RMET Valences

To test whether childhood adversities had any impact on the performance in RMET valences of depressed patients compared with nontraumatized HCs, 3 (group) × 3 (RMET valences: neutral, positive, negative) multivariate mixed-model ANCOVAs were run in each trauma dimension after controlling for age, sex, and years of education.

###### Emotional Maltreatment

*Emotional Neglect (EN).* The first model was a 3 (high-EN MDD, low-EN MDD, and HC groups) × 3 (RMET valences) mixed-model ANCOVA, with age, sex, and years of education as covariates. There was a significant main effect of group (*F*_(2,86)_ = 4.32, *p* = 0.013, η^2^ = 0.09), but the interaction of valence and group did not approach significance (*F*_(4,172)_ = 1.03, *p* = 0.36, η^2^ = 0.02). Pairwise comparisons showed a significant difference between HC and high-EN MDD (*p* = 0.047) (**Table 4**).

**Table 4:** The effect of specific childhood adversities on the response accuracy in RMET valences: results of 3(group) × 3(valence) mixed ANCOVAs.

	The main effect of group		Group x valence interaction	
	*F*_(df1, df2)_η^2^	η^2^	*F*_(df1, df2)_η^2^	η^2^
**EN**	***F*** **_(2,86)_** = 4.32*	**0.09**	F_(4,__172)_ = 1.03	0.02
after controlling for:				
**PA**	***F*** **_(2,85)_** = 3.22*	**0.07**		
**SA**	***F*** **_(2,85)_** = 3.18 *	**0.07**		
PN	*F*_(2,85)_ = 0.74	0.02		
EA	*F*_(2,85)_ = 1.01	0.02		
				
**EA**	***F*** **_(2,86)_** = 5.24**	**0.11**	*F*_(4,__172)_ = 2,92*	0.06
after controlling for:				
**PA**	***F*** **_(2,85)_** = 4.17*	**0.09**	***F*** **_(4,170)_** = 4.79**	**0.1**
**SA**	***F*** **_(2,85)_** = 3.97*	**0.09**	***F*** **_(4,170)_** = 3.91*	**0.08**
PN	*F*_(2,85)_ = 1.45	0.03	*F*_(4,170)_ = 2.17	0.05
EN	*F*_(2,85)_ = 1.18	0.04	*F*_(4,170)_ = 2.35	0.05
				
**PN**	*F*_(2,86)_ = 5.54**	0.11	*F*_(4,__172)_ = 2.01	0.05
after controlling for:				
**PA**	***F*** **_(2,85)_** = 4.46*	**0.1**		
**SA**	***F*** **_(2,85)_** = 4.25*	**0.09**		
EA	*F*_(2,85)_ = 2.61	0.06		
EN	*F*_(2,85)_ = 2.41	0.054		
				
PA	*F*_(2,86)_ = 2.09	0.05	*F*_(4,__172)_ = 1.96	0.04
				
SA	*F*_(2,86)_ = 2.79	0.06	*F*_(4,172)_ = 0.49	0.001

RMET accuracy data were subjected 3(group) × 3(valence) mixed model ANCOVAs with age, sex, and years of educations as covariates. In each analysis, the healthy control group was compared with two MDD groups. Patients with MDD were divided into 2 subgroups on the basis of their scores in CTQ subscales, e.g. MDD with high EA and low EA, etc. Then, other CTQ subscales were entered to control the effect of them. EA, EN, and PN substantially overlap, and mutually extinct the effect of each other on the RMET performance, EN, emotional neglect, EA, emotional abuse, PN, physical neglect, PA, physical abuse, SA, sexual abuse. *p<0.05; ** p<0.01. Effect sizes are presented as η^2^. Statistically significant results are written is bold.

*Emotional Abuse (EA).* To explore the effect of emotional abuse, high-EA MDD, low-EA MDD, and HC groups were entered into the second 3 (group) × 3 (RMET valence) mixed-model ANCOVA with age, sex, and years of education as covariates. There was a significant main effect of group (*F*_(2,86)_ = 5.24, *p* = 0.007, η^2^ = 0.11), and a significant group × valence interaction (*F*_(4,172)_ = 2.92, *p* = 0.023, η^2^ = 0.06). Pairwise comparisons showed a significant difference between HC and high-EA MDD (*p* = 0.008), as well as between high-EA and low-EA MDD groups (*p* = 0.027) (**Table 4**).

**Figure 2:**
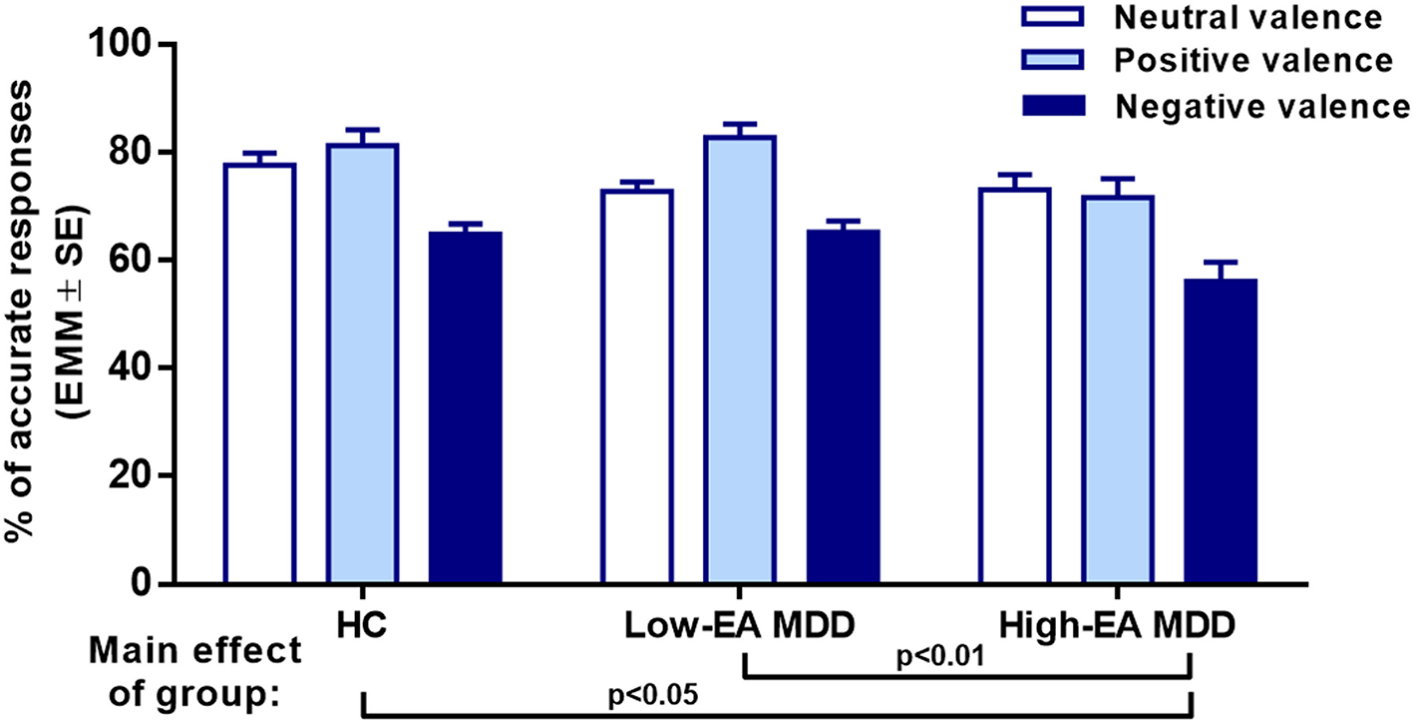
Results of a 3 x 3 mixed ANCOVA with ‘group’ as an independent factor, and RMET valence as a within-subjects factor. Age, sex, and years of education were added as covariates. Estimated marginal means (EMM) are presented. There was a significant main effect of group and a significant interaction between group and valence. EA: Emotional Abuse, HC: healthy control group, Low-EA MDD: a subgroup of MDD patients without moderate or severe childhood emotional abuse, High-EA MDD: a subgroup of MDD patients with moderate or severe childhood emotional abuse.

**Figure 3:**
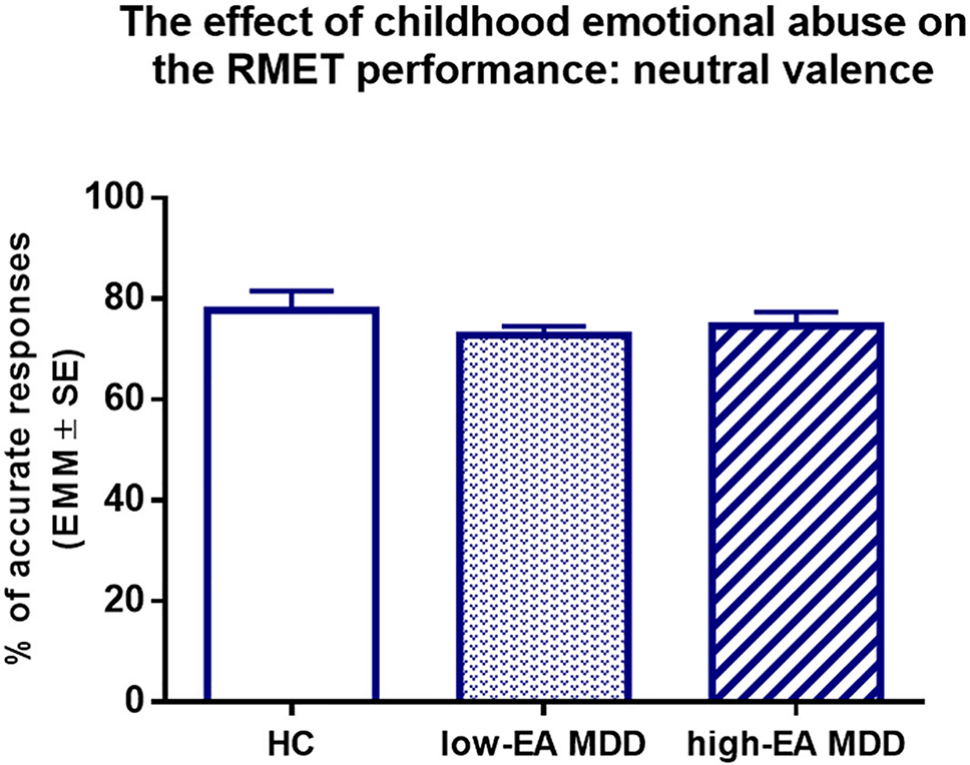
Results of one-way ANCOVA with age, sex, and years of education as covariates. Estimated marginal means (EMM) are presented. There were no significant between-group differences. EA: emotional abuse, HC: healthy control group, Low-EA MDD: a subgroup of MDD patients without moderate or severe childhood emotional abuse, High-EA MDD: a subgroup of MDD patients with moderate or severe childhood emotional abuse.

**Figure 4:**
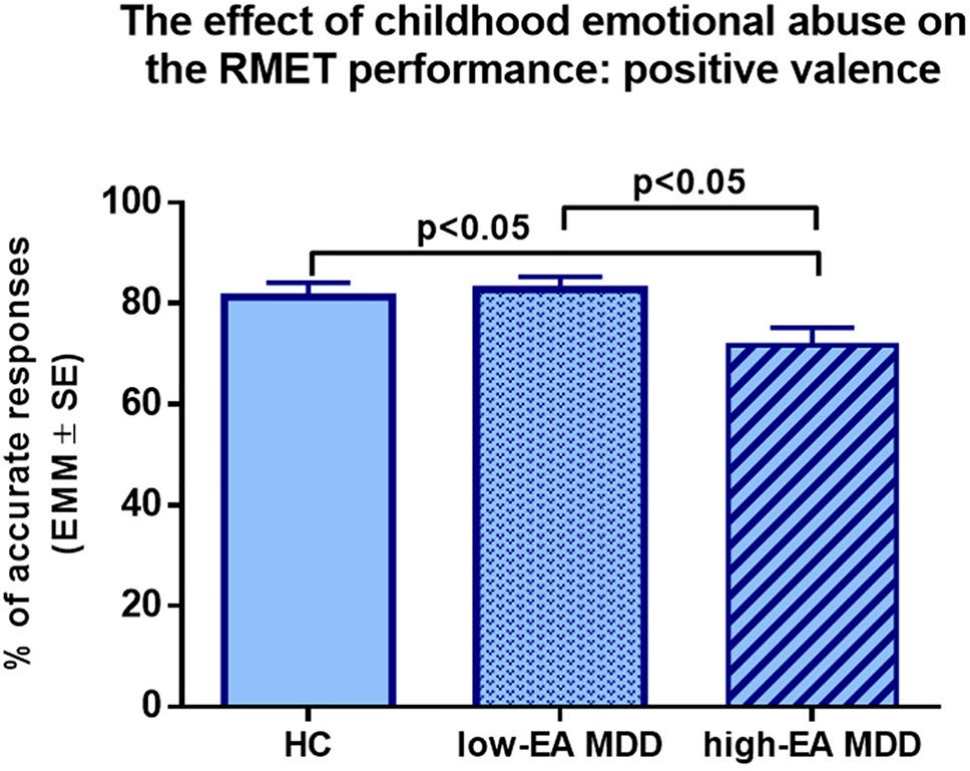
Results of one-way ANCOVA with age, sex, and years of education as covariates. Estimated marginal means (EMM) are presented. MDD patients with moderate or severe childhood emotional abuse performed significantly worse in the positive valence compared to healthy controls and MDD patients without moderate or severe emotional abuse. EA: Emotional Abuse, HC: healthy control group, Low-EA MDD: a subgroup of MDD patients without moderate or severe childhood emotional abuse, High-EA MDD: a subgroup of MDD patients with moderate or severe childhood emotional abuse.

**Figure 5:**
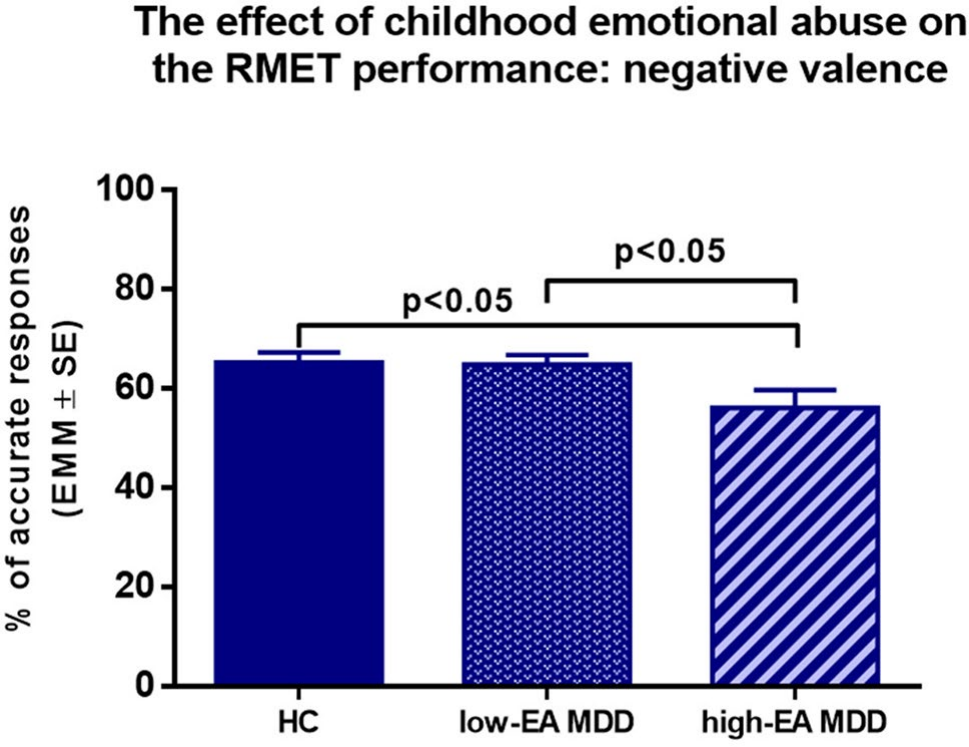
Results of one-way ANCOVA with age, sex, and years of education as covariates. Estimated marginal means (EMM) are presented. MDD patients with moderate or severe childhood emotional abuse performed significantly worse in the negative valence compared to healthy controls and MDD patients without moderate or severe emotional abuse. EA: Emotional Abuse, HC: healthy control group, Low-EA MDD: a subgroup of MDD patients without moderate or severe childhood emotional abuse, High-EA MDD: a subgroup of MDD patients with moderate or severe childhood emotional abuse.

Subsequently, one-way ANCOVAs were conducted by valence with age, sex, and years of education as covariates. In the negative valence, the main effect of group was significant (*F*_(2,86)_ = 3.84, *p* < 0.05, η^2^ = 0.082) (**Figure 2**), *post hoc* Tukey’s multiple comparison test found that the high-EA MDD group significantly underperformed the HC in the RMET (*p* < 0.05). In addition, the low-EA MDD group had a significantly better response accuracy than the high-EA MDD group in the negative valence (*p* < 0.05). Similarly, in the positive valences, there was a significant main effect of group (*F*_(2,86)_ = 5.19, η^2^ = 0.108), *post hoc* Tukey’s revealed that both HC (*p* < 0.05) and low-EA MDD (*p* < 0.01) patients had greater response accuracy than the high-EA MDD group. However, there were no significant between-group differences in neutral valence (**Figures 3–5**).

###### Physical Maltreatment and Sexual Abuse

*Physical Neglect (PN)*. Three (group) × 3 (RMET valence) mixed-model ANCOVA was performed with age, sex, and years of education as covariates. There was no significant group × valence interaction (*F*_(4,172)_ = 2.01, *p* = 0.095, η^2^ = 0.05), whereas a significant main effect of group (*F*_(2,86)_ = 5.54, *p* = 0.005, η^2^ = 0.11) could be detected. Pairwise comparisons showed that MDD patients with high-PN generally underperformed MDD patients with low-PN and HCs (*p* = 0.02 and *p* = 0.004, respectively) in RMET accuracy (**Table 4**).

*Physical Abuse (PA)*. Three (group: HC, low-PA and high-PA) × 3 (RMET valence) mixed-model ANCOVA with age, sex, and years of education as covariates did not reveal any significant group × valence interaction (*F*_(4,172)_ = 1.96, *p* = 0.103, η^2^ = 0.04), or any main effect (*F*_(2,86)_ = 2.09, *p* = 0.13, η^2^ = 0.05).

*Sexual Abuse (SA)*. Only 12 MDD patients reported sexual abuse during their childhood, and all of them were at least moderately emotionally neglected. 91.7% of them were emotionally abused, 50% of them experienced physical neglect, and 33% physical abuse. Three (group: HC, high-SA, and low-SA MDD) × 3 (RMET valence) mixed-model ANCOVA with age, sex, and years of education as covariates yielded only a trend level significance of the main effect of group (*F*_(2,86)_ = 2.79, *p* = 0.067, η^2^ = 0.06), and no group × valence interaction (*F*_(4,172)_ = 0.49, *p* = 0.995, η^2^ = 0.001).

##### Dose–Response Relationship Between the “Number of Traumas” and RMET Inaccuracies in the Entire MDD Group

To test the hypothesis that RMET inaccuracies are a function of the number of childhood adversities in the entire MDD group, three-stage hierarchical multiple regression analyses were conducted with RMET total scores, as well as with RMET scores in neutral, positive, and negative valences. Demographical variables (age, sex, and years of education) were entered at stage one. At stage two the severity of depression (measured with BDI) was entered, followed by “number of traumas” at stage 3.

###### Model 1

First, **RMET total scores** were entered as outcome variables. The best-fitting model for predicting RMET total scores were a linear combination of demographic variables (β_age_ = –0.08, *t* = –0.72, *p* = 0.477; β_sex_ = 0.35, *t* = 2.82, *p* = 0.007; β_EDU_ = 0.21, *t* = 1.722; *p* = 0.091), BDI score (β_BDI_ = –0.14; *t* = –1.14, *p* = 0.258), and number of traumas (β_NTRAUMAS_ = –0.305, *t* = –2.52, *p* = 0.015). Addition of the “number of traumas” did significantly improve prediction (*R* ^2^ change = 0.09, *F*_(1,54)_ = 5.56, *p* < 0.022) (**Table 5**).

**Table 5:** Summary of hierarchical regression analysis for variables predicting RMET total score.

Variable	*B*	SE	β	*t*	*R*	*R* *^2^*	*?R* *^2^*
**Model 1: RMET total**							
***step1***					0.37	0.14	0.14
age	-0.12	0.15	-0.1	-.8			
gender	6.23	2.83	0.28	2.2*			
years of education	1.13	0.5	0.29	2.29*			
***step2***					0.41	0.11	0.03
age	-0.12	0.15	-0.1	-.82			
gender	7.05	2.88	0.31	2.45*			
years of education	1.0	0.5	0.26	2.0			
BDI	-0.38	0.29	-0.17	-1.32			
***step3***					0.5	0.25	0.09
age	-0.10	0.14	-0.08	-0.72			
gender	7.74	2.78	0.35	2.82**			
years of education	0.83	0.49	0.21	1.72			
BDI	-0.31	0.28	-0.74	-1.14			
No. of traumas	-1.42	0.6	-0.31	-2.52*			

N, 60, * p<.05; ** p<.01;

BDI, Beck Depression Inventory score; RMET, Reading the Mind in the Eyes Test, total, total scores.

###### Model 2

Response accuracies in **RMET neutral valences** were entered as outcome variables. Predictive variables were the same as in Model 1. The best-fitting model was a linear combination of demographic variables (β_age_ = –0.17, *t* = –1.4, *p* = 0.168; β_sex_ = 0.25, *t* = 2.02, *p* = 0.048; β_EDU_ = 0.36, *t* = 2.91; *p* = 0.005). Adding BDI, as well as the number of traumas, did not significantly improve prediction (*R* ^2^ change = 0.05, *F*_(1,55)_ = 3.64, *p*< 0.062; *R* ^2^ change = 0.005, *F*_(1,54)_ = 0.35, *p* < 0.556, respectively) (**Table 6**).

**Table 6:** Summary of hierarchical regression analyses for variables predicting RMET scores.

Variable	*B*	SE	β	*t*	*R*	*R* *^2^*	*?R* *^2^*
**Model 2: ** **RMET neutra**l							
***step1***					0.43	0.18	0.18
age	-0.24	0.17	-0.17	-1.4			
sex	6.73	3.33	0.25	2.02*			
years of education	1.69	0.58	0.36	2.91**			
***step2***					0.48	0.23	0.05
age	-0.25	0.17	-0.17	-1.44			
sex	8.11	3.33	0.3	2.43**			
years of education	1.47	0.58	0.31	2.54**			
BDI	-0.64	0.33	-0.24	-1.91			
***step3***					0.49	0.24	0.01
age	-0.24	0.17	-0.17	-1.4			
sex	8.24	3.38	0.31	2.47*			
years of education	1.43	0.6	0.3	2.41*			
BDI	-0.62	0.34	-0.23	-1.84			
No. of traumas	-0.27	0.73	-0.07	-0.59			
							
**Model 3: ** **RMET positive**							
***step1***					0.18	0.03	0.03
age	0.22	0.27	-0.11	-0.82			
sex	5.24	5.09	0.14	1.03			
years of education	0.47	0.89	0.07	0.53			
***step2***					0.18	0.03	0.00
age	0.22	0.27	0.11	0.82			
sex	5.06	5.26	0.12	0.96			
years of education	0.50	0.91	0.08	0.55			
BDI	0.09	0.53	0.23	0.16			
***step3***					0.26	0.07	0.03
age	0.24	0.27	0.12	0.89			
sex	5.81	5.25	0.15	1.11			
years of education	0.32	0.92	0.05	0.35			
BDI	0.16	0.53	0.04	0.3			
No. of traumas	-1.53	1.13	-0.2	-1.35			
							
**Model 4: ** **RMET negative**							
***step1***					0.23	0.05	0.05
age	-0.18	0.23	-0.1	-0.76			
sex	6.21	4.49	0.18	1.38			
years of education	0.82	0.78	0.14	1.06			
***step2***					0.25	0.06	0.01
age	-0.18	0.24	-0.1	-0.76			
sex	6.96	4.62	0.21	1.51			
years of education	0.71	0.8	0.12	0.88			
BDI	-0.35	0.46	-0.1	-0.75			
***step3***					0.45	0.2	0.14
age	-0.15	0.23	-0.08	-0.67			
sex	8.38	4.32	0.25	1.94			
years of education	0.36	0.75	0.06	0.48			
BDI	-0.21	0.43	-0.06	-0.49			
No. of traumas	-2.88	0.93	-0.39	-3.08**			

N, 60, *p<.05; **p<.01; BDI, Beck Depression Inventory score; RMET, Reading the Mind in the Eyes Test, neutral, scores in the neutral valence; positive, scores in the positive valence; negative, scores in the negative valence.

###### Model 3

Next, response accuracies in **RMET positive valences** were entered in the hierarchical regression analysis as an outcome variable. Predictive variables were the same as in Models 1 and 2. Here, none of the predictors contributed significantly to the regression model (**Table 6**).

###### Model 4

Finally, response accuracies in the **RMET negative valences** were subjected to a hierarchical regression analysis as an outcome variable, whereas predictive variables were identical with those in previous models. Only stage 3, the addition of the number of traumas to the regression significantly improved prediction (*R* ^2^ change = 0.14, *F*_(1,54)_ = 9.5, *p*< 0.01), the “number of traumas” explained 38.7% of the variation in response accuracy in RMET negative valences, and it was the only significant predictor of the RMET performance in the negative valence (**Table 6**).

## Discussion

### Main Findings

There was no difference in the mindreading abilities of the healthy controls and MDD patients. However, when we divided the MDD group into two subgroups, one with high and another with low levels of childhood adversities then, a significant difference emerged between the controls and the highly maltreated MDD subgroup in RMET performance. The main finding of our study is that MDD patients with at least moderate childhood neglect or emotional abuse are impaired in the RMET. Furthermore, MDD patients with childhood emotional abuse were significantly less accurate compared to healthy controls and MDD patients without emotional abuse in both the positive and negative valences of RMET. Finally, we found a dose-response relationship between the “number of traumas” and the RMET total scores and the scores of the RMET’s negative valence.

A part of our results is in accordance with the findings of Rnic and co-workers (29), who also investigated the effect of childhood adversities on RMET in MDD. Similar to our present data, Rnic and co-workers found that the history of childhood emotional abuse in patients with MDD significantly worsened the RMET response accuracy. However, our study design differed from that of Rnic and co-workers because we examined only nontraumatized healthy participants. Perhaps due to our rigorous clinical assessment methods, we could identify only four HC individuals who were at least moderately traumatized and had no clinical or subclinical symptoms.

In contrast to the findings of Rnic and co-workers (29), we report here that both emotional and physical neglect had a negative impact on the patients’ RMET performance. In our study, childhood adversities were assessed with CTQ which measures emotional neglect and physical neglect separately. The internal consistency of the physical neglect subscale was the lowest among the subscales, but it was still good (Cronbach’s alpha = 0.77). In general, the exploration and assessment of early-life neglect are relatively difficult, as any kind of neglect has a hidden character (41). On the other hand, emotional neglect strongly intercorrelated with emotional abuse in our sample. As they almost entirely overlapped, it was not possible to investigate their effects separately. **Table 4** shows that the significant effects of both emotional and physical neglect disappeared after controlling for emotional abuse. Hence, we should carefully interpret and generalize our results with the effect of emotional abuse and neglect. Nonetheless, we can conclude that patients who were exposed to both emotional abuse and neglect performed significantly worse in the decoding of subtle emotional cues. To resolve controversies, we applied another approach and included the number of co-occurring types of early-life adversities (= “number of traumas”) for each patient in the MDD group. We found a dose-response relationship between the “number of traumas” and RMET scores: multiple adversities during childhood had a negative effect on RMET total performance and on the performance in the negative valence.

### The Impact of Depression Severity on the RMET Accuracy

Lee and co-workers (8) reported that severely depressed MDD patients’ mental state decoding abilities were significantly worse than those of a healthy community sample, while patients with a mild/moderate MDD did not differ from that. In another study, patients with severe, psychotic MDD significantly underperformed the less severely depressed, nonpsychotic MDD patients in ToM tasks (10). While an additional study investigating less severely depressed individuals found that patients in a major depressive episode performed more accurate than healthy controls in the negative emotional valence of RMET (12). Furthermore, increased sensitivity to social stimuli was observed in a population sample with dysphoria (measured with BDI): a college sample with dysphoria had superior accuracy in decoding mental states compared to a nondysphoric group (7). Greater accuracy in RMET performance was reported in depressed and nondepressed women with a maternal history of depression (1), and in patients with past MDD (11) indicating that both an increased vulnerability for depression and a previous depressive episode can significantly enhance the capacity to accurately identify subtle emotional cues.

Our findings are in harmony with the results reviewed above. We examined a relatively homogeneous group of MDD patients with mild or moderate depression, and we rigorously controlled the HC group for subclinical symptoms. When we compared the entire MDD group with HC, we found no significant between-group differences in RMET performances. However, significant between-group differences appeared when we analyzed the MDD subgroup with a high level of early adversities (= high-ACE MDD group) separately. Moreover, BDI scores were entered into our final hierarchical regression model. The “number of traumas” significantly predicted the inaccuracies in RMET total scores and in the negative valence even after controlling for severity of current depression. Hence, childhood adversities were much stronger predictors of RMET inaccuracies than the severity of depression in our mildly/moderately depressed MDD group.

### The Effect of Early-Life Adversities: Possible Underlying and Mediating Factors

Exposure to childhood adversities (particularly to emotional abuse) is known to result in increased reactivity of the amygdala which makes the affected individuals more vulnerable to negative psychosocial experiences during adulthood (42). In addition, early-life stress results in dysregulation of the hypothalamus-pituitary gland-adrenal axis, which makes the individual more susceptible to stressful life situations (43,). Thus, we can assume, that negative social cues evoke increased stress, particularly in those MDD patients who were exposed to emotional abuse (e.g. anger, hostility) as a child, which might worsen their mental state decoding abilities particularly in the emotional valences. These assumptions are in line with the results of Hentze and co-workers (45) who investigated chronically depressed patients’ regional brain activations during an affective ToM task and found significantly increased amygdala activation in patients with childhood maltreatment. Altough no brain-imaging study has examined the brain activations during ToM tasks specifically in adult MDD patients with early adversities (46), we can speculate that MDD patients who were exposed to early life stress might react with a more pronounced amygdalar activation that might impair their RMET accuracy.

A recent study, measuring the peak amplitudes of N170 face-sensitive visual ERP component responses to emotional faces in asymptomatic adults with or without childhood trauma, suggested that exposure to childhood trauma was associated with a failure to differentiate between threat-related and nonthreat-related emotional stimuli (47). These findings indicate that early-life stress may be related to a generalized emotional hyper-responsibility to emotional cues, which in turn makes it difficult to recognize accurately the emotional content of them (47). Moreover, it was found that early-life parental maltreatment (particularly physical abuse but also neglect to a certain extent) negatively influenced RMET accuracy in a large, internet-based, adult community sample (17). Hence, we can assume, that the emotional hyper-reactivity and the difficulties of emotional regulation—due to the increased amygdala activity—make it more challenging for asymptomatic individuals with early adversities to mirror adequately the emotional contents and identify them. In a recent functional MRI study, a regional hyperactivation of the pars triangularis was detected in youth with sexual abuse and emotional maltreatment during the RMET task, which was interpreted as a compensation of the impaired mirroring capacities of the participants (48). In addition, the authors concluded that the combination of sexual abuse and emotional maltreatment can be particularly toxic. This corresponds with our results on the negative effect of the co-occurrence of various maltreatment types on RMET.

### Clinical Relevance of the Findings

MDD patients with childhood adversities have an elevated risk of developing recurrent or chronic depressive episodes, and are more often therapy-resistant (e.g., meta-analyzed by 49–51). According to our findings, early-life emotional abuse, mostly if they co-occurred with physical and emotional neglect resulted in impaired mental state decoding capacities in MDD. In particular, the decoding of emotional cues (both negative and positive) was inaccurate.

Considering that early-maltreated MDD patients have a reduced ability to decode social cues, one can predict that they experience significantly higher levels of stress in social situations, especially when they need to assess emotional cues. This may worsen their social withdrawal, as well as increase their existing vulnerability to stressful life events. All these are in harmony with the common clinical and epidemiological experience that early-life adversities worsen the outcome and the course trajectory of MDD. In sum, when planning their therapy, clinicians need to consider the effect of childhood adversities, they should count on therapy resistance or chronicity, and should strive to remediate these patients’ ToM and mentalizing abilities.

### Limitations of the Study

The major limitation of our study was that no never-depressed individuals with at least a moderate degree of abuse or neglect were entered in the analysis. On the basis of the literature, we aimed to control the effect of subclinical depressive symptoms, therefore, inclusion criteria were relatively strict. The very few HC subjects with relevant childhood adversities (N = 4) were excluded from the analysis because their number was too low to analyze them as a separate group. Further analyses are necessary to test the effect of adverse childhood experiences on the mental state decoding abilities in healthy individuals, to disentangle the effect of MDD and that of childhood adversities. Hence, we should very carefully interpret our findings on the role of childhood adversities.

Furthermore, childhood emotional abuse, emotional neglect, and physical neglect were associated with inaccurate RMET performance in MDD patients, whereas no effects were observed for physical and sexual abuse. Nevertheless, the prevalence of physical and sexual abuse was relatively low in our MDD sample. As a consequence of it, the differential effects of specific types of early life adversities might also be a consequence of differential statistical power. In addition, childhood adversities were measured retrospectively, during an acute depressive episode. Therefore, recall biases induced by the current depression could not be fully ruled out (52).

Another limitation is that we did not include the Beck Anxiety Inventory (BAI) scores in our multiple regression models. The severity of anxiety and depression strongly overlapped in our MDD sample (Spearman’s rho = 0.613, *p* < 0.001), so we interpreted it as a symptom of the current depression. Nevertheless, our primary correlation analyses revealed a strong correlation between BAI scores and the severity of childhood adversities (rho = 0.341, *p* < 0.008). Anxious depression is a common subtype of MDD among patients with childhood adversity. It has been recently reported that anxious depression in patients with early-life adversities sensitizes the glucocorticoid receptors (53).

The effects of neurocognitive functions and verbal abilities were not extensively examined in our study. (We assessed IQ, VQ, and PQ). Nevertheless, we found that the total RMET response accuracy and that in the neutral valence correlated with the years of education (Pearson’s r = 0.323, *p* < 0.001; and r = 0.411, *p*< 0.001, respectively). There was a weaker, but still significant correlation (Pearson’s r = 0.262, *p* < 0.05; r = 0.305, *p*< 0.01, respectively) between IQ and RMET performance as well. Although Baron-Cohen and co-workers (6) reported that the intelligence did not contribute to the processes involved in RMET, a recent meta-analysis involving 77 studies found a small positive correlation (r = 0.24) between IQ and RMET performance without any significant difference between the effects of verbal and performative intelligence (54). In addition, several studies found a correlation between executive functions and ToM capacities in MDD (meta-analyzed by 4). In the future, more extensive assessment of neurocognitive functions, verbal fluency, and vocabulary are necessary to evaluate their exact impact on RMET accuracy, especially on that in the neutral valence, as well as the way in which childhood adversities can influence it.

Finally, we did not control the effect of antidepressant medication. The majority of our clinical MDD sample (95%) was medicated and took antidepressants from various classes. Therefore it was not possible to form homogeneous medication groups for further analysis.

## Conclusions

In sum, our present findings document that childhood adversities impair the mental state decoding abilities of MDD patients during the acute episode. Multiple adversities in general, but particularly the co-occurrence of emotional abuse and neglect, were found to have a more disadvantageous effect with a dose-response character on mental state decoding capacities in MDD. Further (preferably follow-up) research is needed to clarify the exact underlying mechanisms and therapeutic interventions beneficial for this subgroup of MDD patients.

## Data Availability Statement

All datasets generated for this study are included in the article/**Supplementary Material**.

## Ethics Statement

The local Research Ethics Committee of the University of Pécs approved the study design and protocol (Ethical Approval Nr.: 2015/5626) and all participants provided written informed consent

## Author Contributions

MS, BC, and NN conceived and designed the study. NN, EC, EL, and MG collected the data. Data analysis was performed by MS with special assistance from NN, EC, MG, and EL. MS wrote the manuscript. TT provided supervision and had helpful comments on the interpretation of the data. All authors listed have made a substantial, direct and intellectual contribution to the work, and approved it for publication.

## Funding

This work was financially supported by the following grant agencies: Hungarian Brain Research Program (KTIA_NAP_13-2-2014-0019 and 20017-1.2.1-NKP-2017-00002), EU Social Funds (EFOP-3.6.3-VEKOP-16-2017-00009, EFOP-3.6.2-16-2017-00008, “The role of neuro-inflammation in neurodegeneration: from molecules to clinics”) and by The Higher Education Institutional Excellence Programme of the Ministry of Human Capacities in Hungary, within the framework of the 20765-3/2018/FEKUTSTRAT 5^th^ thematic program of the University of Pécs. These grant agencies had no influence on study design; in the collection, analysis, and interpretation of data; in the writing of the report; and in the decision to submit the article for publication.

## Conflict of Interest

The authors declare that the research was conducted in the absence of any commercial or financial relationships that could be construed as a potential conflict of interest.
