# Supplementary material for: Childhood Adversity Impairs Theory of Mind Abilities in Adult Patients With Major Depressive Disorder
Source: Front Psychiatry. 2019 Dec 17;10:867. doi: 10.3389/fpsyt.2019.00867 (PMC6928114; doi:10.3389/fpsyt.2019.00867)
Supplement: Supplementary file 1 [file DataSheet_1.docx]

***Supplementary Material***

**Childhood adversity impairs theory of mind abilities in adult patients with major depressive disorder**

**Authors:**

Maria Simon^1,2,*^, Nándor Németh^1^, Mónika Gálber^1^, Elza Lakner^1^, Tamás Tényi^2^, Boldizsár Czéh^1,3^

*** Correspondence:** Maria Simon, MD, PhD

Department of Psychiatry and Psychotherapy, University of Pécs, Medical School,

H-7623 Pécs, Rét u. 2., Hungary

E-mail: [simon.maria@pte.hu](mailto:simon.maria@pte.hu)

**Table 1.**

Correlations between RMET scores and clinical and demographical variables within the whole sample

|  | RMET total | RMET neu | RMET pos | RMET neg | Age | years of  EDU |
| --- | --- | --- | --- | --- | --- | --- |
| CTQ PN | -.204 | -.049 | -.169 | -.214* | -.074 | -.191 |
| CTQ PA | -.220* | -.077 | -.118 | -.269** | .025 | -.026 |
| CTQ EN | -.214* | -.113 | -.098 | -.151 | .142 | -.242* |
| CTQ EA | -.234* | -.095 | -.166 | -.131 | -.044 | -.283** |
| CTQ SA | -.176 | -.217* | .044 | -.037 | .145 | -.276** |
| CTQ total | -.232* | -.127 | -.116 | -.159 | .059 | -.284** |
| Age§ | .005 | -.118 | .087 | .213* | -.119 |  |
| years of EDU§ | .323** | .398** | .098 | .052 | .075 |  |
| HAM-D | -.253* | -.242* | -.109 | -.066 | -.134 |  |
| BDI§ | -.287** | -.318** | -.063 | -.02 | -.111 |  |
| BAI | -.231* | -.256* | -.047 | -.032 | -.075 |  |

N=92. Spearman’s rhos are presented. § Pearson’s correlation coefficients are presented.

RMET= Reading the Mind in the Eyes Test; total= total score, neutral= neutral valence; pos= positive valence, neg= negative valence; EDU= years of education; HAM-D= Hamilton Rating Scale for Depression, BDI= Beck Depression Inventory, BAI= Beck Anxiety Inventory, length= length of MDD (years). CTQ= Childhood Trauma Questionnaire, PN= physical neglect, PA=physical abuse, EN= emotional neglect, EA= emotional abuse, SA= sexual abuse, total= total scores; **p*<0.05, ***p*<0.01

**Table 2.**

Results of Spearman’s correlations between RMET performances and CTQ scores in the MDD group.

|  | RMETsum | RMETneu | RMETpos | RMETneg |
| --- | --- | --- | --- | --- |
| No of traumas | -0,294* | -0,052 | -0,198 | -0,362* |
| CTQPN | -0,230 | -0,020 | -0,201 | -0,292* |
| CTQPA | -0,207 | 0,011 | -0,004 | -0,392* |
| CTQEN | -0,192 | -0,024 | -0,146 | -0,195 |
| CTQEA | -0,213 | -0,023 | -0,209 | -0,180 |
| CTQSA | -0,170 | -0,263* | 0,052 | -0,026 |
| CTQsum | -0,220 | -0,050 | -0,146 | -0,232 |

Spearman’s rhos are presented. * = *p*< 0.05

**Table 3.**

Correlations between RMET scores, early adversities (A), and clinical variables (B)within the entire MDD group.

**A)**

|  | CTQ  physical neglect | CTQ  physical abuse | CTQ  emotional neglect | CTQ  emotional abuse | CTQ  sexual abuse | CTQ  total score |
| --- | --- | --- | --- | --- | --- | --- |
| age at onset | -.044 | -.174 | -.199 | -.323* | -.282* | -.237 |
| length of MDD | .036 | .401** | .13 | .141 | .104 | .152 |
| No. episodes | .132 | .214 | .437** | .385** | .360** | .381** |

**B)**

|  | RMET  total | RMET  neutral | RMET  positive | RMET  negative |
| --- | --- | --- | --- | --- |
| age at onset | -.108 | -.127 | -.04 | -.091 |
| length of MDD (years) | -.468** | -.300* | -.225 | -.374** |
| No. episodes | -.171 | -.236 | .026 | -.085 |

Spearman’s rhos are presented. N=60. **p*<0.05, ***p*<0.01

RMET= Reading the Mind in the Eyes Test; total= total score, neutral= neutral valence; pos= positive valence, neg= negative valence; CTQ= Childhood Trauma Questionnaire

CTQ scores strongly correlated with variables of the course of the MDD, as well as with RMET scores (Table 1).

**Table 4.**

As expected, patients with MDD scored significantly higher on depression and anxiety scales. Patients with MDD had significantly higher scores in all subscales and total score of the CTQ, and ETI.

Childhood trauma and parental bonding scores of the participants:

|  | **HC**  **N=32** | **MDD**  **N=60** | **Statistic** |
| --- | --- | --- | --- |
| ***CTQ total scores and subscales*** |  |  |  |
| CTQ sum | 30.5 (27-34) | 49.5 (33-64.75) | *U*=313*** |
| CTQ PN | 5 (5-6) | 7.5 (5-10) | *U*=380.5*** |
| CTQ PA | 5 (5-6) | 5.5 (5-9) | *U*=568** |
| CTQ EN | 8 (6-11) | 14 (10-18.75) | *U*=317*** |
| CTQ EA | 6.5 (6-8) | 10 (7-18.75) | *U*=390.5*** |
| CTQ SA | 5 (5-5) | 5 (5-6) | *U*=579.5** |
| ***ETI total scores and subscales*** |  |  |  |
| ETI GEN | 2 (0-2) | 2 (1-4) | *U*=709.5* |
| dysfunctional family | 0 (0-1) | 1 (0-2) | *U*=573.0*** |
| ETI PA | 1 (0-1.75) | 1 (0-3) | *U*=789.5 |
| ETI EA | 0 (0-2) | 2 (0.25-4.75) | *U*=562*** |
| ETI SA | 0 (0-0) | 0 (0-1) | *U*=565.0*** |

HC= healthy Control group; MDD= Major Depressive Disorder group; CTQ= childhood Trauma Questionnaire; PN= physical neglect; PA= physical abuse; EN= emotional neglect; EA= emotional abuse; SA= sexual abuse; ETI= Early Trauma Inventory, GEN= general trauma score; dysfunctional family= dysfunctional family items, *U*= Mann-Whitney *U*;

Because of the lack of the normal distribution medians (interquartile intervals) are presented; **p*<0.05; ***p*<0.01; ****p*<0.001

**Table 5.**

Results of bivariate correlations between IQ, and years of education, as well as RMET total, neutral, negative and positive scores. Since IQ, and the years of education strongly inter-correlated, but the years of education had a stronger association with all RMET data, the years of education were entered to further analysis

Correlation analyses between RMET scores and IQ, and years of education:

|  | IQ | years of education |
| --- | --- | --- |
| RMET total scores | *0.262 | ***0.359 |
| RMET neutral valence | **0.305 | ***0.411 |
| RMET positive valence | 0.014 | 0.074 |
| RMET negative valence | 0.176 | 0.205 |
| years of education | ***0.723 |  |

Note. Pearson’s correlation coefficients are presented. N=92.

**p*<0.05; ***p*<0.01; ****p*<0.001.

Cells with significant correlation coefficients are highlighted.

RMET= Reading the Mind int he Eyes Test

**Table 6.**

Correlations between CTQ subscales.

|  | CTQ  physical abuse | | CTQ  emotional neglect | CTQ  emotional abuse | CTQ  sexual abuse | |
| --- | --- | --- | --- | --- | --- | --- |
| CTQ physical neglect | **0.492 | | **0.759 | **0.737 | **0.356 | |
| CTQ physical abuse |  | | **0.572 | **0.642 | **0.309 | |
| CTQ emotional neglect |  |  | | **0.851 | **0.418 |  |
| CTQ emotional abuse |  |  | |  | **0.503 |  |

Note. CTQ= Childhood Trauma Questionnaire. Spearman’s rhos are presented. N=92.

Rhos > 0.6 are highlighted. ** Correlation is significant at the *p*< 0.01 level.

**Table 7.**

Overview of the frequency of the various forms of childhood adversities across the 4 degrees of severity in the MDD group. In our study, the prevalence of retrospectively reported childhood maltreatment harmonized with data in the literature: emotional traumas (neglect and abuse) were most commonly reported by patients with MDD followed by neglect.

Frequency of the specific types of abuse and neglect in the overall MDD group (N=60):

|  | None;  No. of  cases (%) | Low;  No. of  cases (%) | Moderate;  No. of cases (%) | Severe;  No. of cases (%) | **No. of at least moderately maltreated MDD patients (%)** |
| --- | --- | --- | --- | --- | --- |
| CTQ emotional neglect | 13 (21.7) | 20 (23.3) | 8 (13.3) | 19 (31.7) | **27 (45)** |
| CTQ emotional abuse | 11 (18.3) | 14 (13.3) | 3 (5) | 19 (31.7) | **22 (36.7)** |
| CTQ physical neglect | 33 (55) | 12 (20) | 9 (15) | 6 (10) | **15 (25)** |
| CTQ physical abuse | 43 (71.7) | 5 (8.3) | 6 (10) | 6 (10) | **12 (20)** |
| CTQ sexual abuse | 45 (75) | 7 (11.7) | 9 (15) | 3 (5) | **12 (20)** |

CTQ= Childhood Trauma Questionnaire. The numbers of cases (%) are presented.

**Table 8.**

Correlations between CTQ, and ETI scores.

|  | CTQ PN | CTQ PA | CTQ EN | any neglect | CTQ EA | CTQ SA | CTQ total |
| --- | --- | --- | --- | --- | --- | --- | --- |
| ETI GEN | 0.486** | 0.394** | 0.470** | 0.393** | 0.511** | 0.315** | 0.536** |
| dysfun.family | 0.668** | 0.449** | 0.677** | 0.590** | 0.676** | 0.414** | 0.724** |
| ETI PA | 0.415** | 0.674** | 0.427** | 0.353** | 0.543** | 0.376** | 0.536** |
| ETI EA | 0.657** | 0.682** | 0.692** | 0.622** | 0.778** | 0.468** | 0.767** |
| ETI SA | 0.414** | 0.236* | 0.514** | 0.523** | 0.482** | 0.792** | 0.515** |
| ETI total | 0.668** | 0.673** | 0.695** | 0.582** | 0.762** | 0.481** | 0.775** |

CTQ = Childhood Trauma Questionnaire; PN= physical neglect, PA= physical abuse, EN= emotional neglect, EA= emotional abuse, SA= sexual abuse; ETI= Early Trauma Inventory; GEN= general trauma subscale; dysfunc.family= dysfunctional family items; PBI= Parental Bonding Instrument, m= mother, f= father, over= ’overprotection’ subscale, cont= ’control’ subscale.

Pearson’s correlation coefficients are presented. **p*<0.5, ***p*<0.01; ****p*<0.001.

**Table 9.**

Differences between ***HC*** and ***MDD*** groups (Analysis of Covariance with age, sex, and years of education as covariates) in the Reading the Mind in the Eyes Test total scores and valences.

| Source | df | MS | F | *p* | η^2^ |
| --- | --- | --- | --- | --- | --- |
| RMET total score |  |  |  |  |  |
| Covariate age | 1 | 119.78 | 1.52 | 0.221 | .017 |
| Covariate sex | 1 | 236.27 | 2.99 | 0.087 | .033 |
| **Covariate years of EDU** | **1** | **858.06** | **10.87** | **0.001** | **.111** |
| Group | 1 | 259.69 | 3.29 | 0.073 | .036 |
| Error | 87 | 78.92 |  |  |  |
| Total | 92 |  |  |  |  |
|  |  |  |  |  |  |
| RMET neutral score |  |  |  |  |  |
| Covariate age | 1 | 330.48 | 2.65 | 0.108 | .030 |
| Covariate sex | 1 | 66.51 | 0.53 | 0.468 | .006 |
| **Covariate years of EDU** | **1** | **2039.17** | **16.32** | **0.000** | **.158** |
| Group | 1 | 378.76 | 3.03 | 0.085 | .034 |
| Error | 87 | 124.95 |  |  |  |
| Total | 92 |  |  |  |  |
|  |  |  |  |  |  |
| RMET positive score |  |  |  |  |  |
| Covariate age | 1 | 447.68 | 1.76 | 0.189 | .020 |
| **Covariate sex** | **1** | **1801.81** | **7.06** | **0.009** | **.075** |
| Covariate years of EDU | 1 | 166.75 | 0.65 | 0.421 | .007 |
| Group | 1 | 255.13 | 1.00 | 0.320 | .011 |
| Error | 87 | 255.13 |  |  |  |
| Total | 92 |  |  |  |  |
|  |  |  |  |  |  |
| RMET negative score |  |  |  |  |  |
| Covariate age | 1 | 456.23 | 2.5 | 0.118 | .028 |
| Covariate sex | 1 | 56.98 | 0.31 | 0.578 | .004 |
| Covariate years of EDU | 1 | 377.55 | 2.07 | 0.154 | .023 |
| Group | 1 | 168.58 | 0.92 | 0.340 | .010 |
| Error | 87 | 182.78 |  |  |  |
| Total | 92 |  |  |  |  |

HC= healthy control, MDD= major depressive disorder, EDU= education.
